# Light Trapping with Silicon Light Funnel Arrays

**DOI:** 10.3390/ma11030445

**Published:** 2018-03-19

**Authors:** Ashish Prajapati, Yuval Nissan, Tamir Gabay, Gil Shalev

**Affiliations:** 1 Department of Electrical and Computer Engineering, Ben-Gurion University of the Negev, POB 653, Beer-Sheva 8410501, Israel; ashish@post.bgu.ac.il (A.P.); nissay@post.bgu.ac.il (Y.N.); tamirgab@post.bgu.ac.il (T.G.); 2 The Ilse-Katz Institute for Nanoscale Science and Technology, Ben-Gurion University of the Negev, POB 653, Beer-Sheva 8410501, Israel

**Keywords:** light trapping, photovoltaics, solar cells, light-funnel arrays, nanophotonics, photon management, mode excitation

## Abstract

Silicon light funnels are three-dimensional subwavelength structures in the shape of inverted cones with respect to the incoming illumination. Light funnel (LF) arrays can serve as efficient absorbing layers on account of their light trapping capabilities, which are associated with the presence of high-density complex Mie modes. Specifically, light funnel arrays exhibit broadband absorption enhancement of the solar spectrum. In the current study, we numerically explore the optical coupling between surface light funnel arrays and the underlying substrates. We show that the absorption in the LF array-substrate complex is higher than the absorption in LF arrays of the same height (~10% increase). This, we suggest, implies that a LF array serves as an efficient surface element that imparts additional momentum components to the impinging illumination, and hence optically excites the substrate by near-field light concentration, excitation of traveling guided modes in the substrate, and mode hybridization.

## 1. Introduction

The interaction of light and matter and specifically the coupling of light into matter is of both scientific and technological interest. Light trapping is about capturing photons from an incident electromagnetic wave, normally in the range from the infrared to the ultra-violet. Surface texturing with ordered or disordered arrays with subwavelength (or wavelength-scale) features has been demonstrated to increase light trapping in thin films (TF) beyond the Yablonovitch limit [1,2,3,4,5] Furthermore, surface arrays with subwavelength features are an additional strategy for the development of ultra-thin photovoltaic cells. Ultra-thin solar cells with absorption comparable to bulk solar cells directly lead to lower recombination currents and higher open circuit voltages, and therefore to higher photovoltaic efficiencies [6], as well as allowing the commercialization of photovoltaic cells based on scarce materials. 

Surface texturing with ordered or disordered tiling of subwavelength features has been shown to enhance the broadband absorption of the solar radiation due to light trapping (e.g., vertically-aligned nanopillars (NPs), nanoholes (NHs), rods, nanocones (NCs), nanospheres, etc.) [2,3,5,7,8,9,10,11,12,13,14,15,16,17,18,19,20]. Note that in the current context, NPs refer to diameters of several hundred nanometers. In a planar semiconducting film, for example, both radiation and trapped traveling modes (guided modes and Bloch modes) are present [21]. However, the wavenumbers of the guided modes (i.e., photonic states) are not accessible to the radiation impinging on the top surface unless some extent of scattering or diffraction takes place. The Yablonovitch limit assumes ‘mixing of the light’ inside the absorber medium by randomizing the texture of the top and bottom interfaces, and in this manner generates wavenumbers that can occupy both radiation and guided modes and hence maximize light trapping. Arrays of subwavelength structures can impart wavenumber additions to the impinging photons by diffraction and/or scattering, and render the various guided modes available for occupation [22,23,24]. In addition, arrays of semiconducting subwavelength structures introduce additional modes to the system in the form of localized trapped modes (or Mie modes) residing inside the subwavelength structures, and also by hybridization of Mie modes with the other available modes [7]. 

Absorption enhancement with semiconducting subwavelength vertically-aligned NP arrays has been demonstrated [3,17,25,26,27,28,29,30,31,32,33,34,35,36,37,38,39,40,41,42,43]. It was shown that the decoration of the surfaces of solar cells with subwavelength structures–specifically with NP arrays–confers on those cells a light trapping capacity that exceeds the Yablonovitch limit [2,3,4,5]. Fountaine et al. numerically demonstrated near unity broadband absorption by GaAs sparse NP arrays [17], and recently, they showed (experimentally) near unity absorption in InP NP arrays [37]. Wallentin et al. demonstrated 13.8% photovoltaic efficiency with InP NP arrays by exceeding the ray optic limit [3]. Spinelli et al. demonstrated black silicon with arrays of silicon nanocylinders for which the absorption was attributed to forward scattering by the cylinders [30]. We numerically demonstrated that high broadband absorption of the solar spectrum with silicon NPs is optimally achieved with an array period of ~500 nm and NP diameter of ~400 nm [44], and we recently discussed strategies for efficient carrier collection from optimized nanopillar arrays [45] and minority carrier collection from NP-substrate complexes [46].

Another promising family of structures consists of periodic or randomized vertical subwavelength cone arrays [36,47,48], the incorporation of which in solar cells introduces a gradual refractive index profile and promotes favorable optical impedance matching between the textured surface and the ambient. Huang et al. reported reflectance of below 1% for silicon nanotip arrays with a tip height of 16 µm [49]. Jeong et al. reported a record efficiency for a thin silicon solar cell of 13.7%, which was attributed to enhanced absorption due to surface nanocone arrays [50]. Most recently, a record efficiency of 22.1% was reported for black silicon [51]. 

In a recent publication, we introduced the light funnel array, which is a new light trapping scheme bio-inspired by the *fovea centralis* [52]. With LFs, we refer to subwavelength cones that are inverted with respect to incoming illumination (as opposed to the upright NCs described above). The *fovea centralis* is a closely-packed vertical array of inverted-cone photoreceptor cells located in the retina that is responsible for high acuity binocular vision under bright light conditions, and in this sense it resembles the functionality of a photovoltaic (PV) cell. In the very same publication, we presented a numerical three-dimensional (3D) finite-difference time-domain (FDTD) study of theoretical free-floating silicon LF arrays (height of 2 µm and no substrate) and showed that LF arrays can be realized. Moreover, we numerically demonstrated that the broadband light absorption of the solar spectrum in free-floating LF arrays is superior to the absorption in optimized NP arrays, and furthermore, it is superior to other outstanding recent advancements in the field (nanocylinder and nanocone arrays [30,50,53]). The absorption of the free-floating LF arrays was compared with that of optimized free-floating NP arrays (500 nm period and 400 NP diameter). We demonstrated that by decreasing the bottom diameter of the optimized NP (i.e., the formation of a LF), the ultimate absorption efficiency (*η_ult_*) of the array increased by ~65% relative to the continuous film in comparison with NP arrays that present an absorption enhancement of 36.6%. Enhanced angular response (angle of incident) was also demonstrated. Additionally, the absorption of the free-floating LF array is higher despite the smaller filling ratio (22% for the LF array and 50% for the pillar array). Furthermore, we showed that the NPs in free-floating NP arrays exhibit low order modes, whereas the free-floating LFs exhibit complex 3D modes; at a specific wavelength, the hypothetical deformation of the NP into a LF modifies the constellation of internal reflections inside the cavity and renders the formation of complex 3D modes (trapped localized modes). The presence of complex 3D modes provides a strong optical coupling between the incoming radiation and the free-floating LF arrays that is manifested in distinct absorption peaks.

In the following, we present a study of the optical coupling between LF arrays and underlying substrates and, in particular, we explore the optical excitation of the substrate by the LF arrays. 

## 2. Materials and Methods

We employed a 3D FDTD optical simulation using Advanced TCAD by Synopsis (Mountain View, CA, USA). The simulation box size was set to the size of the unit cell, with a periodic boundary condition along the lateral dimensions. The bottom boundary condition was defined by the gold back reflector. The periodic boundary condition was applied to the normally incident plane wave excitation using the total-field scattered-field (TFSF) formulation. Both the magnetic and electric fields were copied directly from the periodic facet to the opposing one during field update. For each run (each wavelength), absorption and reflection were calculated using sensors that were located above the device (no transmission was recorded on account of the gold bottom reflector). In addition, for each wavelength the power flux density and the absorbed photon density at each mesh point were calculated. The absorbed photon density was calculated simply by dividing the absorbed power density ($\frac{1}{2}$ × *σ* × |*E*^2^| in which *σ* is the nonzero conductivity of silicon and *E* is the impinging electric field) by the energy of the impinging photon. TM polarization was used, and the various LF cross-sections showing the absorbed photon density or the power flux density were normal to the plane of incidence. The calculations were performed in the spectrum range of 400–1000 nm in 20 nm steps. For efficient and accurate FDTD simulations, the maximum mesh cell size was kept smaller than 1/10th of the wavelength in silicon; namely, more than 10 nodes per wavelength. The ultimate absorption efficiency (*η_ult_*) is the relative absorption averaged and weighted with the solar spectrum, and where it is assumed that each photon above the bandgap generates an electron–hole pair that is collected at the electrodes. The *η_ult_* was calculated in the following manner:

(1) $$\eta_{ult}=\frac{\int_{E_{g}}^{\infty }I\left(E\right)A\left(E\right)\frac{E_{g}}{E}\;dE}{\int_{0}^{\infty }I\left(E\right)dE}$$

where, $E_{g}$ = 1.1 eV is the bandgap of silicon, *E* is the photon energy, *A*(*E*) is absorption spectrum and *I*(*E*) is the solar irradiance taken under Air Mass 1.5 Global (AM 1.5G) conditions. The optical constants of silicon material were taken from the literature [54]. 

## 3. Results

**Figure 1:**
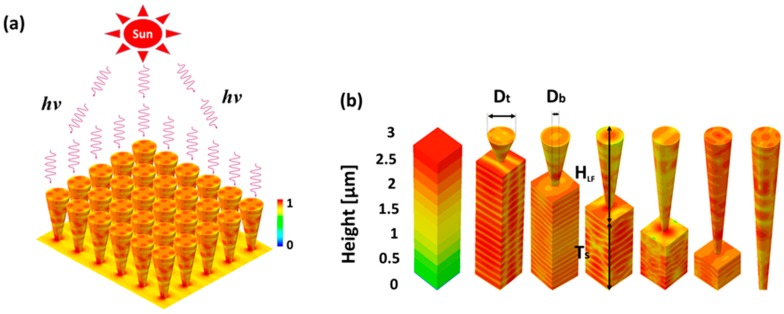
The LF array-substrate complex. (**a**) An illustration of an LF array on top of a substrate. The illumination is from above. The LF array is an infinite square-tiled array, where the individual color-coded LFs reflect the normalized absorbed photon density. (**b**) A schematic describing the various relevant dimensions associated with the complex, and the various geometries considered in the current study. The color coding reflects the simulated normalized absorbed photon density. The simulations reflect the various possible optical excitations (the images were obtained for different wavelengths and are not scaled).

Figure 1a presents an illustration of a 3D silicon LF array on top of a substrate. The color coding reflects the normalized absorbed photon density (a certain arbitrary wavelength was selected for the illustration); still, note the formation of higher-order complex modes at the top of the LFs (quadrupole) and the lower-order modes apparent at the bottom interface between the LFs and the substrate (dipole), which reflect near-field light concentration (or forward scattering) by the LF array into the substrate. Figure 1b shows individual LFs on top of various substrates in which the full height of the LF array-substrate complex is maintained (3 μm) but the ratio between the LF height (*H_LF_*) and the substrate thickness (*T_s_*) varies; hence, the considered *H_LF_s* are 0, 0.5, 1, 1.5, 2, 2.5 and 3 μm (*T_s_* is adjusted such that the total height of the LF array-substrate complex is 3 μm). Note that the 3 μm thickness of the LF array-substrate complex was arbitrarily selected as a study case. In the current examination, we assume a fixed LF top diameter (*D_t_*) of 400 nm and a fixed LF bottom diameter (*D_b_*) of 100 nm. The array period (*P*) is set to 500 nm, as it was demonstrated for NP arrays that 500 nm periodicity couples best to the solar spectrum, as the solar spectrum peaks at around this wavelength [44]. In the present work, the LF array geometry was not optimized to maximize the absorption of the solar radiation; rather, we consider the deformation of an optimized NP array into a LF array. Therefore, it is most probable that the absorption of the LF array could be further enhanced. The images in Figure 1b are 3D FDTD results for different geometries at certain wavelengths, and the color coding describes the normalized absorbed photon density, which reflects the various mode excitations in the LF array-substrate complex. It is evident from Figure 1b that the presence of LF arrays on top of a substrate concludes various excitations of optical modes both in the LF array and in the substrate. Furthermore, in order to enhance the optical coupling between the LF arrays and the substrate, we consider in the following a conformal 50 nm SiO_2_ anti-reflective coating (ARC) decorating the top of the LF array-substrate complex (in practice, a conformal ARC could be a challenge for the inverted LF arrays. However, a conformal ARC could be realized using atomic layer deposition (ALD), producing conformal thin layers regardless of the surface topography) and a gold reflector at the bottom of the substrate (neither the ARC or the gold reflector are shown in Figure 1). Finally, note that the thickness of the ARC was not optimized.

**Figure 2:**
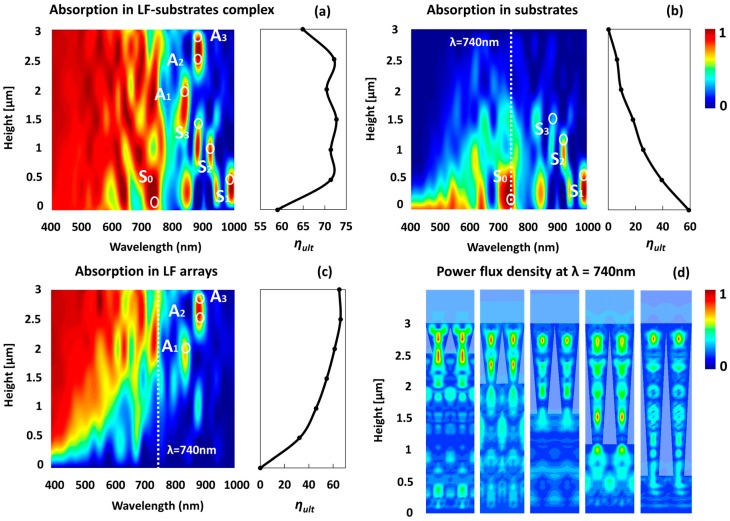
(**a**) The relative absorption of the LF array-substrate complex for the various geometries. The respective *η_ult_* are shown on the right. (**b**) The relative absorption of the substrate component of the LF array-substrate complex for the various geometries. The respective *η_ult_* of the substrates are shown on the right. The color-coded scale bar in Figure 1b is for Figure 2a–c. (**c**) The relative absorption of the LF array component of the complex for the various geometries. The respective *η_ult_* of the LF arrays are shown on the right. (**d**) The normalized power flux density at wavelength 740 nm. Note the various optical excitations in the substrates as well as the LFs the near-field light concentration into the substrates. The cross-sections are normal to the plan of incidence.

Figure 2a presents a color map of the simulated relative absorption spectra of the 3 μm LF array-substrate complex for various LF heights (and the respective substrate thicknesses that conclude the 3 μm complex). The respective *η_ult_* of each spectrum (i.e., for each geometry) is plotted on the right. The bottom of the color map reflects the relative absorption in a 3 μm TF (i.e., no LF array at all), whereas the top-most spectrum in the color map presents absorption in a 3 μm LF array (i.e., no substrate at all). Evidently, the *η_ult_* of the 3 μm LF array is ~14% higher than the *η_ult_* of the 3 μm TF. Note that in reference [52], the *η_ult_* of the LF arrays is significantly higher than the *η_ult_* of the thin film. This is because in the current study, we consider a gold bottom reflector, and, as expected, the gold bottom reflector substantially increases the absorption in TFs. Moreover, the gold bottom reflector of the 3 μm LF array is restricted to the LF bottom diameter (i.e., bottom reflector with a diameter of 100 nm), whereas for the thin film, the gold reflector extends throughout the bottom of the simulated unit cell (i.e., throughout the bottom of the film). Still, in the current study, we consider the presence of gold bottom reflector as our current aim is to explore the optical coupling between the LF arrays and the substrates and particularly the optical excitation of the substrates by the LF arrays. To this end, the presence of the gold bottom reflector is assumed, as it inevitably amplifies the optical interaction between the arrays and the substrates. For the 3 μm LF array, the broadband light absorption is attributed to efficient light trapping associated with mode hybridization of localized trapped optical modes (Mie modes) and Fabry-Perot (FP) modes, which are generated due to the bottom gold reflector. For the 3 μm thin film the absorption is due to light trapping associated with FP radiation modes. Interestingly, note that the *η_ult_* of the 3 μm LF-substrate complex is always higher than the *η_ult_* of the 3 μm LF array (~10%). This suggests an efficient optical coupling between the LF arrays and the substrates and, moreover, an efficient optical excitation of the substrate by the LF array. The presence of the substrate introduces additional photonic states in the form of traveling guided modes such as waveguide modes and Bloch modes and hybridization of these (and with FP). Overall, it is evident that although the LF arrays host a high density of complex Mie modes that conclude efficient light trapping and light absorption, the LF array-substrate complex still offers a superior system for light trapping, as the LF array excites various modes (and hybridizations) in the substrate in addition to the conventional radiation modes. 

It is evident in Figure 2a that the *η_ult_* of the LF array-substrate complex depends only weakly on the ratio between *H_LF_* and *T_s_*. Figure 2b,c show the decoupling of the relative absorption spectrum of the LF array-substrate complexes into the relative absorptions of the substrate and the LF array, respectively. As expected, the higher the LF arrays are, the higher the absorption in the arrays is (Figure 2c), and similarly, the absorption in the substrate increases for smaller LFs and thicker substrates. Decoupling the contributions of the substrates and the LF arrays to the overall absorption of the complex reveals the origin of the strong absorption peaks evident in Figure 2a. For example, note the absorption peaks in Figure 2a marked in S0–S3 and A1–A3. The formation of the S0–S3 absorption peaks is attributed to strong excitations in the substrate (note the marked absorption peaks in Figure 2b), and the formation of the A1–A3 absorption peaks is traced to strong excitations in the LF arrays (note these same peaks in Figure 2c). Importantly, note that the A1–A3 absorption peaks occur at wavelengths smaller than 900 nm, whereas absorption peaks S1 and S2 occur at wavelengths exceeding 900 nm. This suggests that the proposed geometries, when engineered properly, can induce strong optical excitation of the substrate at the near infra-red (NIR) which is of great interest for thin-film photovoltaics, for example. 

Figure 2d presents the normalized power flux density at wavelength 740 nm (marked in dashed white line in Figure 2b,c) for the selected geometries. The excitation of various optical modes and mode hybridization in the LF arrays and in the substrates is apparent, as well as forward scattering (or near-field light concentration) of the LF arrays into the substrates, which is present in every geometry. Note that at wavelength of 740 nm, the LF arrays are strongly excited; still, the overall contribution of the arrays to the absorption is considerably smaller for short arrays, as is evident, for example, for the LF array of 0.5 μm and the substrate of 2.5 μm, in which the LF array is highly excited but the overall absorption in the array is small compared with the absorption in the substrate. 

**Figure 3:**
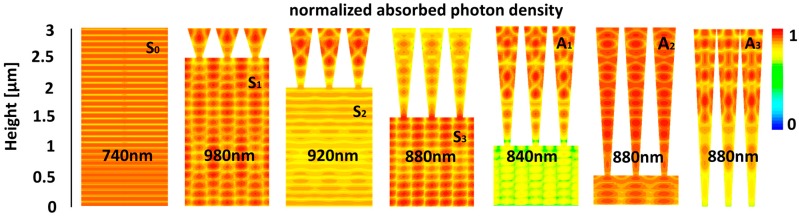
3D FDTD results of the normalized absorbed photon density for various geometries and wavelengths. The S0–S3 and A1–A3 notations refer to Figure 2a–c. The cross-sections are normal to the plan of incidence.

Figure 3 presents the normalized absorbed photon density for different geometries and wavelengths pertaining to the A1–A3 and S0–S3 absorption peaks marked in Figure 2a–c with white circles. Firstly, note the different excitation mechanisms that are responsible for the strong absorption peaks. The strong absorption of the TF at S0 is due to FP modes. The strong absorption at S1 is due to the hybridization of FP modes and guided modes in the substrate. In S2, the strong absorption is also due to strong excitation of the substrate but in this case FP modes govern the excitation. In S3 the absorption is due the strong hybridization of FP and traveling guided modes, whereas the excitation of Mie modes in the array is minor despite the considerable height of the array. Finally, the A1–A3 absorption peaks are governed by strong hybridization of FP and Mie modes in the arrays. 

## 4. Conclusions

In the current work we study light trapping in an LF array-substrate complex. We show that the broadband light absorption is higher in LF array-substrate complexes compared with the broadband absorption in an LF array (without a substrate) of the same height. The absorption enhancement is attributed to the generation of additional momentum components to the impinging illumination, which results in near-field light concentration by the LF array and mode excitation and mode hybridization in the substrate. Finally, we show that the ratio between the height of the LF array and the thickness of the substrate has little effect over the broadband absorption of the solar spectrum by the complex.
